# New Appliances and Things Medical

**Published:** 1899-12-09

**Authors:** 


### NEW APPLIANCES AND THINGS MEDICAL.

[We shall be glad to receive, at our Office, 28 & 29, Southampton Street, Strand, London, W.C., from the manufacturers, specimens of all new preparations and appliances which may be brought out from time to time.]

### CHINOSOL AND ITS PREPARATIONS.

(The Chinosol Hygienic Co., 36, St. Mary-at-Hill, London, E.C.)

Chinosol has now such an established reputation as an antiseptic that little need be here added with respect to its merits. Since being introduced to the public some six years ago it has steadily won its way in popularity. It is perhaps in the treatment of diseases of the throat, ear, and nose that up to the present it has found its most useful province. Surgeons and medical men are peculiarly conservative in their methods, and in no way do they demonstrate this tendency more than in the use of antiseptics. Take the case of carbolic acid, for instance: this is almost the invariable medium which is employed for the cleaning of instruments before and after use. Its properties are, of course, undoubted as an antiseptic, and, moreover, it has no destructive influence on metals, but it has a disagreeable odour, damages the hands, and is not nearly such a convenient or powerful antiseptic as many others. Tradition, however, dies hard, and carbolic acid will continue to be used with all its inconveniences. Chinosol, which belongs to the quinoline group, is a most powerful antiseptic, is most conveniently carried in the form of tablets, and has almost universal application. Recently the Chinosol Hygienic Company has perfected a new soap in which chinosol has been incorporated. For surgical and other uses it should be invaluable, whether for toilet purposes or for the bath, for which it is supplied in suitable form and strength. For disinfecting on a large scale chinosol is supplied in the crude form. For sanitary and municipal authorities this is of value, supplying a magnificent antiseptic in a cheap form.

### CARNIGEN.

(Leete and O'Connell, St. Dunstan's Buildings, St. Dunstan's Hill, London, E.C.)

Carnigen is a new meat preparation in a desiccated form. It is a fine powder, which to a considerable extent is soluble in water. The solution is to be compared to raw meat juice in its chemical and clinical properties. The powder, whether in solution or suspension, is extraordinarily assimilable, and clinical experience seems to show that even in pathological conditions with which loss of weight is usually associated the loss may be checked, or eplaced by a gain, by the use of this new preparation. Carnigen is practically tasteless, and well tolerated by the stomach even in irritable conditions. It has probably a useful future before it.

